# Iterative co-creation for improved hand hygiene and aseptic techniques in the operating room: experiences from the safe hands study

**DOI:** 10.1186/s12913-017-2783-1

**Published:** 2018-01-04

**Authors:** Annette Erichsen Andersson, Maria Frödin, Lisen Dellenborg, Lars Wallin, Jesper Hök, Brigid M. Gillespie, Ewa Wikström

**Affiliations:** 1 Institute of Health Care Sciences, Sahlgrenska Academy, University of Gothenburg, Box 457, 405 30 Gothenburg, Sweden; 2 Sahlgrenska University Hospital, Gothenburg, Sweden; 3 School of Education, Health, and Social Studies, Dalarna University, Falun, Sweden; 4 Department of Neurobiology, Care Sciences and Society, Division of Nursing, Karolinska Institutet, Solna, Sweden; 5 GPCC Implement, University of Gothenburg, Gothenburg, Sweden; 6 School of Nursing and Midwifery, Griffith University, Nathan, Australia; 7 Gold Coast University Hospital and Health Service, Southport, Australia; 8 School of Business, Economics and Law, Department of Business Administration, University of Gothenburg, Gothenburg, Sweden

**Keywords:** Aseptic technique, Co-creation, Implementation, Knowledge translation, Hand hygiene, Interprofessional learning, Operating room

## Abstract

**Background:**

Hand hygiene and aseptic techniques are essential preventives in combating hospital-acquired infections. However, implementation of these strategies in the operating room remains suboptimal. There is a paucity of intervention studies providing detailed information on effective methods for change. This study aimed to evaluate the process of implementing a theory-driven knowledge translation program for improved use of hand hygiene and aseptic techniques in the operating room.

**Methods:**

The study was set in an operating department of a university hospital. The intervention was underpinned by theories on organizational learning, culture and person centeredness. Qualitative process data were collected via participant observations and analyzed using a thematic approach.

**Results:**

Doubts that hand-hygiene practices are effective in preventing hospital acquired infections, strong boundaries and distrust between professional groups and a lack of psychological safety were identified as barriers towards change. Facilitated interprofessional dialogue and learning in “safe spaces” worked as mechanisms for motivation and engagement. Allowing for the free expression of different opinions, doubts and viewing resistance as a natural part of any change was effective in engaging all professional categories in co-creation of clinical relevant solutions to improve hand hygiene.

**Conclusion:**

Enabling nurses and physicians to think and talk differently about hospital acquired infections and hand hygiene requires a shift from the concept of one-way directed compliance towards change and learning as the result of a participatory and meaning-making process. The present study is a part of the Safe Hands project, and is registered with ClinicalTrials.gov (ID: NCT02983136). Date of registration 2016/11/28, retrospectively registered.

## Background

A crucial challenge in contemporary healthcare is the ability to provide effective, evidence-based care that minimizes the risk of painful and costly iatrogenic harm and adverse events. National Swedish data indicate that hospital-acquired infections (HAI) are still the most common and most costly preventable complication related to surgical care [1]. Approximately 10% of all patients are affected by one or more HAI, and the reduced availability of effective treatments makes the situation even more serious [2]. Implementing solutions to combat HAI and antimicrobial resistance is a complex process that requires multimodal methods including the judicious use of antimicrobial agents and comprehensive infection prevention measures. Thus, leaders and health professionals are tasked with the challenge of translating research evidence into practice and implementation of new or revised practices, which imposes pressure on organizational development and change, learning, and quality of care [3].

As hand hygiene (HH) is regarded one of the most important strategies to prevent HAI and the spread of microorganisms [4–8], finding effective interventions to ensure its sustained use in healthcare is of the utmost importance. The situation is considered so crucial that in 2005 the World Health Organization launched an initiative to improve HH practice to save patients’ lives [9], including the publication of comprehensive guidelines [10]. Nevertheless, in clinical environments where the risks for transmission are high and patients are more susceptible to infection, adherence to recommended practice is reported to be lower (30–40%) than in other settings (50–60%) [11]. The lowest compliance rates (2–18%) have been reported in the operating room (OR) context during anesthetic care [12–15]. Recent work has shown that lack of appropriate HH poses a serious risk for patients due to cross-contamination, the spread of microorganisms, and postoperative infections [16–18]. However, there is a paucity of research into interventions aimed at improving HH practices in the OR context [19, 20].

The most common HH interventions in other settings typically consist of education, reminders, feedback, and access to alcohol-based hand rub [21, 22]. Still, these types of bundled strategies are not always successful; improvements are usually small to moderate and little is known about mechanisms of change. A systematic review of HH interventions [20] found that studies were often of poor quality. Interventions were rarely described in sufficient detail, and the context and theoretical underpinnings of the intervention strategies were often omitted. Thus, the positive or negative outcomes derived from these interventions are difficult to explain. There is a growing acknowledgment that the absence of process evaluation in the implementation of complex interventions impedes not only replication but also the generation of important knowledge on the effectiveness of different implementation strategies [23, 24].

The literature and major implementation frameworks suggest the importance of considering the characteristics of the innovation and the context, including culture and leadership, when introducing change processes [25–27]. It is important to assess barriers and enablers prior to an intervention [28]. A multitude of studies have investigated observed and self-reported barriers to HH among different professional groups and in different settings [11, 14, 29–31]. Contextual patient safety barriers related to the OR have been described, with much attention paid to deficits in interpersonal relationships [32–34], role perceptions [35], communication patterns, and teamwork [32, 36–39], and the negative impact on the safety of patients in surgery [33, 40, 41]. The OR context has been defined as a high-risk and complex environment, where specialized nurses, physicians, and sometimes technicians work together under capricious conditions [42]. Surgical teams are often assembled ad hoc, and the members work together for only short periods of time [43].

An understanding of how nurses, physicians, and managers perceive, interact with, and respond to a knowledge translation (KT) intervention can provide insight into the intervention’s acceptability, feasibility, and likelihood of a deeper impact on the OR culture. It is equally important to understand how the characteristics of the innovation, the professionals and managers, and the context interrelate and influence the response to, and the delivery of, the KT program, as these interactions will most likely impact the adoption in practice and over the longer term, improve patient safety. The approach taken by the facilitators and their interactions with the participants and the context likely influences the KT process, and thus contribute to a better understanding of what works and what does not. This study aimed to evaluate the process of implementing a theory-driven KT program to improve the use of HH and aseptic techniques (AT) in the OR. Within this aim we sought to explore and describe the process of co-creation of the content and activities of the intervention between participants and facilitators. The overarching goals of the Safe hands study was to increase awareness, knowledge and ownership of postoperative infections (i.e., surgical site infections and device-related infections) within different professional groups in surgery.

## Methods

### Design

**Fig. 1:**
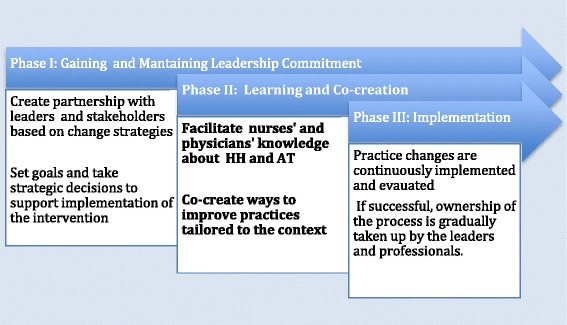
An overview of the three phases of the Knowledge Translation program

An prospective design based on ethnographic fieldwork [44] and structured observations was used to evaluate the process of implementation. The present study is a part of the Safe Hands project, and is registered with ClinicalTrials.gov (ID: NCT02983136). The project consists of three overlapping phases and will result in a series of companion publications. Findings regarding HH performance and infectious outcome after hip fracture surgery will be published with reference to the present publication. In this article, we report process findings derived from phase II, lasting from February to December 2016, see Fig. 1.

### Participants and setting

The study setting was an orthopedic OR department in performing approximately 10,000 orthopedic surgeries/year. The department consists of 7 ORs and employs 127 professionals including certified operating room nurses, nurse anesthetists, anesthesiologists, and nurse assistants. The OR is also staffed with 25 specialists in orthopedic surgery. Twelve health professionals, including two from each above-mentioned disciplines participated in the learning laboratories described below. Three of these were first line managers and one middle-manager.

### Tailoring the intervention

The intervention in this study entailed a flexible and emergent KT program. The content of program was co-created with the participants and developed for research purposes. Selection of the theory-informed strategies was based on previously defined barriers related to the implementation objective and the context. In addition, analysis throughout the intervention was used to tailor the methods and strategies to emerging situations and specific organizational and cultural barriers and enablers. One key feature of the KT program was the use of facilitated interprofessional dialogue and the creation of a “safe container for learning” [45, 46] termed *Learning Labs* (Labs). The Labs were hosted by two facilitators with expertise in the OR context and infection control (AEA), and in leadership and change management (JH), respectively. AEA has a background as a certified OR nurse, lecturer and researcher. JH has a background as a social behaviorist and extensive experience of facilitating change process within nonprofit organizations.

Their roles were not to diagnose and describe solutions for change, but rather to facilitate the participants’ learning and problem solving in a complex environment; that is, helping them learn *how* to learn. The program strategies for interactions were based on the ethical assumptions of person centeredness, in which healthcare professionals, like patients, are seen as persons with intentions, capabilities and competent experts on their own realities [47].

### Data collection

Participant observation [48] was conducted by an experienced social anthropologist not involved in the design of the study or delivery of the intervention. The method includes unobtrusive observations of interactions and natural conversations as well as informal interviews; the intent throughout was to keep an open mind and non-judgmental attitude in order to understand organizational and cultural behavior from within. The data consisted of field notes collected over 121 h during all Labs, workplace meetings, and informal talk in the OR. The purpose of using participant observations was to document the implementation process and to gain a deeper understanding of how culture and leadership interacted with the KT program and shaped relationships and actions. Structured observation of the participants, including their roles, attendance rates, and hours spent in the Labs, was used to collect data on fidelity to the intervention [24, 49].

### Data analysis

Data derived from participant observations were analyzed using a thematic approach [50]. Initially, all the field notes were transcribed by the anthropologist and then read by three co-authors to gain a sense of the whole. In the first cycle of coding, the data were organized according to areas of content, followed by condensation of meaning units and the creation of inductive codes. In the second cycle of analysis, pattern coding was used to seek similarities, differences, and relationships in the material. This process resulted in a summarization of the data under themes and subthemes. The data were independently analyzed by three of the researchers, after which the themes and subthemes were discussed among all researchers until consensus was reached. Data derived from structured observations were analyzed by manifest content analysis [51].

## Results

The findings are structured under three headings: *Intervention delivery*, *Fidelity to the intervention*, and *The complexity and emergence of knowledge translation.*

### Intervention delivery

For an overview of the identified and targeted barriers, theory-based implementation strategies, proposed mechanisms of change, and the KT processes used for delivery see Table 1. The process of identifying barriers was ongoing throughout the intervention. The participants were encouraged to talk about difficulties encountered in their everyday work. Different professional groups identified different types of barriers for change. The facilitators could give examples from the litterateur in support of their observations and initiate a discussion on how these barriers could be addressed. These suggestions were linked to theories but the activities selected were co-created by merging the participants knowledge and experiences with facilitators knowledge and experiences.

**Table 1:** Progressively identified and targeted barriers related to the OR context, and their linkages to theory-based strategies, proposed mechanisms of action, and the intervention activities

Targeted barriers	Theory-based KT strategy	Proposed mechanisms of action	KT activities
Lack of teamwork, trust, and communication	Interprofessional learning [66] and dialogue within a safe learning “container” [45]	Increased levels of interaction Strengthened relations Improved understandings between professionals groups and managers	Facilitating regular dialogue meetings between a selected group of professionals and managers (*the Learning Lab group*)
Lack of knowledge regarding HAI, patient outcome and HH performance	Audit and feedback [60] Education based on adult learning theory [67], situated and experiential learning [68, 69]	Increased motivation and commitment Internal drive for seeking knowledge and change	Visualization of patient outcome data and behavioral feedback Mindful observations of one’s own and one’s peers’ HH practice Facilitated problem-based learning Co-creation of printed information material Reviewing “My five moments for hand hygiene”
Skepticism about the value of HH and AT	“Celebrating” resistance Challenging basic assumptions [46]	Decreased skepticism about the evidence in support of HH and AT Increased commitment to change	Workshop welcome and encouraging participants’ diverse perspectives Facilitators actively seek to understand and address hesitation, questions and resistance in a respectful way Reviewing the evidence for HH and AT in relation to invasive procedures Co-producing printed evidence-based information on HH
Lack of tailoring of the clinical guidelines to the OR context	Co-creation [70, 71] and design thinking [72]	Relevant and meaningful HH and AT routines	Step 1: Welcome and promote innovative new ideas; sense, probe, respond and reflect in an iterative process in the *Learning Lab group* Step 2: Involve OR staff in the testing, reflection and refining of standardized operational procedures
Lack of role models and opinion leaders	Using facilitators [27] with social impact [28]	Role modeling from credible and trusted sources	Strive to create honest relationships between facilitators and participants Lab participant as change agents
Deficits in clinical leadership and change management skills	Facilitating development of clinical leadership skills [46]	Increased ability to understand and manage implementation in complex environments Increased awareness of the importance of leadership in change processes	Interactive mini-lectures on leadership, implementation and change management

The participants in the Labs were selected by their managers in phase I (Fig. 1). The numbers of Labs and workplace meetings were negotiated between managers, participants, and researchers. The initial choice of six Labs finally resulted in 11 two-hour Labs held from January to December 2016. The participants and facilitators also hosted seven workplace-based dialogue meetings with the OR staff, three meetings with orthopedic surgeons, and two meetings with anesthesiologists in order to start the process of overall involvement. Except for the predefined focus on HH and infection control, the activities in the Labs were tailored based on the participants’ needs, their level of knowledge, and their own ideas for activities.

To give an insight into how the work in the Labs evolved, a description of the first Lab is provided with a short summary of the core activities, the participants’ reactions, and the group’s development. Table 2 shows the goals of the first Lab and the central issues raised by the participants. Having the ward manager as a part of the Lab served two purposes: first, to ensure that the ideas and innovations created in the Lab had the support of management, and second, to demonstrate that management not only supported the project but owned it, and was willing to learn together with the other participants.

**Table 2:** The goals of the first Learning Lab, and central issues raised by the participants

The goals of the first learning laboratory and workplace-based meeting
• Open up for dialogue • Create awareness about the problem of the lack of HH and AT in the OR and postoperative infections among patients undergoing hip-fracture surgery. • Start the process of creating a shared sense of urgency within the organization • Learn more about post-operative infections and how to create chance and co-create new knowledge • Clarifying roles, goals, and working methods
Issues and central questions addressed in the learning laboratories, exemplified by quotations
*“Is there really any evidence in support of HH and AT?”* *“How can we involve all our co-workers in the OR?”* *“How to move away from telling someone that they are wrong or failing to see this as an opportunity for learning away from shame and blame?”* *“How can we create awareness around our own practices?”* “*Will this be another project without physician engagement, that will fail?”* *“What is the right way to do it…?”* *“We don’t have the time to talk about or observe each other doing this* [HH] *during work.”*

#### Between the labs

Between the Labs, the participants worked on different assignments including observation of their own practices and involving other colleagues by actively seeking their perspectives and input. After each Lab, the facilitators critically reflected on their own performance, the participants’ narratives, the issues raised, and the problems observed. A strategy for facilitating the next Lab was established on the basis of these reflections. Informal meetings with key stakeholders were held between the Labs, to allow these stakeholders to continue to work on commitment and support. The facilitators assisted participants to make sense of their experiences during everyday work, including difficult or challenging interactions with their colleagues outside the Labs. The mini-lectures emphasized the concepts of culture, complexity, and change management.

### Fidelity to the intervention

A total of 12 professionals participated in the Labs, but none of them attended all 11 Labs. The highest continuity in attendance was seen among the nurses. The median attendance rate was 7, range (0–12). The ninth Lab, which was the first after the summer holidays, was cancelled as no participants showed up. However, the effort to renew a sense of urgency and commitment among front-line leaders resulted in this Lab being re-run at a later date. The importance of front-line leadership support became evident during the study period. When managers were engaged in the Labs, attendance rates increased, and during times when the managers were absent or prioritized other duties, attendance rates dropped off. Discontinuity in the Lab remained an ever-present challenge during the intervention as participants were absent for different reasons including lack of time, the necessity to do other work, understaffing, or having a day off. Physicians’ attendance at the Labs was dependent on how senior managers enabled participation. Pre-planning was decisive, as the Labs had to be booked 6 months in advance to be accommodated into physicians’ work schedules. Information on the participants is given in Table 3.

**Table 3:** Participants and the number of Labs attended

Profession & Role	Attendance rates/participants during 11 Labs
Nurse assistant	8
Nurse assistant	9
OR nurse	10
OR nurse and clinical instructor	7
Nurse anesthetist	6
Nurse anesthetist	10
Anesthesiologist and clinical chief physician	8
Anesthesiologist^a^	6
Orthopedic surgeon^b^ (senior)	4
Orthopedic surgeon (junior)	6
Intensive care nurse and OR ward manager	6
Nurse anesthetist and OR front-line nurse manager	7

^a^One of the anesthesiologists was replaced at the 8th Lab, as he/she had moved to another hospital

^b^The senior orthopedic surgeon experienced difficulties in taking part due to lack of time, and so was replaced at the 8th Lab

### The complexity and emergence of knowledge translation

The qualitative analysis resulted in two main themes and 12 related subthemes; see Table 4 for an overview. The themes and subthemes are described in more detail below to provide insight into how contextual factors interacted and impacted on the content and activities in the intervention, how barriers were addressed, and what worked as a catalyst for change.

**Table 4:** Themes and subthemes showing deterrents to knowledge translation and central aspects of the intervention that worked as catalysts for change

Over-arching theme	Knowledge translation - a complex and emergent process	
Themes	1. Deterrents to knowledge translation	2. Catalysts for learning and change
*Subthemes*	*1.1 Balancing conflicting goals and system ambiguities* *1.2 Unknown patient consequences* *1.3 Doubts that HH and AT prevent HAI* *1.4 Strong boundaries, hierarchies, and distrust.* *1.5 A culture of right and wrong*	*2.1 Facilitation as an iterative process of creating trusting relationships* *2.2 The creation of a shared sense of urgency* *2.3 Co-creation and iterative prototyping* *2.4 A growing awareness of the workplace culture and one’s own practices* *2.5 Increased psychological safety through dialogue*

### Deterrents to knowledge translation

This theme describes the most important hindrances for KT within the OR context.

#### Balancing conflicting goals and system ambiguities

The middle and front-line managers had difficulty balancing the strong demands for increased productivity and shorter turnover times with the demands for safety and quality. Front-line managers expressed concerns that participation in the project would potentially lead to disruption of daily work activities. Thus, the front-line managers were not initially convinced that the Safe Hands project was worthwhile or necessary. In the early stages of the project, some professionals stated that “in reality, safety is subordinate to production” and that “patient safety issues are not taken seriously”. The lack of time for reflection, innovation, and learning was a persistent issue raised during the Labs.

Prior to the intervention, learning activities occurred only within professional groups, and there was no common platform for co-operation or meetings between nurses and physicians. The nursing staff particularly expressed frustration over the lack of opportunities for interprofessional learning. After the intervention, managers regularly scheduled interprofessional meetings and used parts of the KT program strategies in other change projects.

#### Unknown patient consequences

During the study period, several conditions that hampered engagement in learning were observed during the Labs and workplace-based meetings. The general lack of knowledge among professionals regarding infectious complications after surgery had resulted in a false sense of security, and so the drive for change was initially absent.

#### Doubts that HH and AT prevent HAI

A major barrier to learning was the managers’, nurses’, and physicians’ doubts that HH and AT were effective in preventing HAIs. Moreover, several physician participants questioned the evidence around HH relative to preventing the spread of microorganisms and reducing the risk of HAI:

“There’s no evidence that hand hygiene will reduce the risks of infection.”

“Until there’s a double blind RCT study showing this, I’m going to carry on as usual.”

Nursing staff as a group tended to more easily accept the evidence base as sufficient when presented to them by the facilitators. Conversely, the anesthesiologists needed to engage in repeated discussion and debates rather than the intended dialogue. One participant highlighted the issue of the source of the information:

*“If we want to convince the physicians that this is important, they’ll need to hear the evidence from a peer* [rather than from another profession].”

*“We know that you* [the RN/facilitator] *are an expert in this field, but that’s not enough.”*

Actively encouraging expressions of hesitation and the challenging of evidence gave the facilitators the ability to tailor the presentation and discussion of evidence in relation to all the participating professional groups. However, the facilitators were not able to fully create the conditions required for true dialogue within some of the professional groups, due to lack of access and the limited time allocated. Hence, some participants remained hesitant towards change throughout the study period.

#### Strong boundaries, hierarchies, and distrust

Strong boundaries and hierarches between professional groups were identified as hindering learning within and between professional subgroups during everyday work. Nurses’ offers to assist physicians with task-related infection control strategies was sometimes met with defensiveness and resistance, but also with appreciation. The different professional groups often expressed stereotyped understanding of each other’s roles, and little knowledge about each other’s workload and tasks. However, most participants expressed an interest in improving the suboptimal teamwork that seemed to characterize their interprofessional interactions. Nursing staff repeatedly highlighted the lack of mutual understanding, trust, and adequate communication. The lack of trust was not only towards other professions but also between other nursing staff and managers. In addition, disruptive behaviors were not uncommon, and increased the lack of psychological safety between professional groups. This became even more evident during the intervention, as the Lab participants perceived that they had to put themselves in risky situations just by observing and talking about HH and AT with co-workers. In contrast, respectful, helpful, and humorous interprofessional interactions were also observed.

#### A culture of right and wrong

The lack of psychological safety within the workplace, in combination with the intervention that challenged basic assumptions of what was right and wrong and what type of knowledge could be heard and appreciated, created anxiety among some of the participants and made them reluctant to test new practices. Before the intervention, the typical approach when seeing someone doing something “wrong” had been either to point it out in a rather harsh tone or to not say anything at all. The participants were uncomfortable addressing potentially risky behaviors, and found it very difficult to take on an inquiring approach as suggested in the Labs; asking and listening instead of telling. The idea of giving feedback by telling someone *“Don’t do that — it’s wrong”* was so deeply rooted that it was difficult to change this type of behavior. For physicians, it was out of the question to “remind” their colleagues: *“You make sure to keep good relations with your colleagues; that’s just the way it is.”* The dialogue in the Labs revolved around how this culture of right and wrong impacted on behaviors and how new ways of interacting could be tested. Some participants crossed the hierarchical boundaries to become role models who inspired others and learned others’ techniques on how to go about things.

### Catalysts for learning and change

This theme describes the most important aspects of the intervention that worked as drivers to facilitate learning and change.

#### Facilitation as an iterative process of creating trusting relationships

Even though potential barriers to change were identified prior to the intervention, through studies and the literature, the process of facilitation had to be agile and flexible. It was impossible to know beforehand what kind of support and help the participants needed. Through focusing on creating trusting relationships with the Lab participants and the OR staff, the facilitators were able to identify some of the basic assumptions about inter- professional relations, infection control and HH at the deeper levels of the culture, which opened participants up to new ways of thinking. The person-centered approach that guided interactions was important in creating trust between facilitators and participants.

#### The creation of a shared sense of urgency

The presentation and visualization of patient outcome data describing postoperative complications and adverse events following hip-fracture surgery at the clinic contributed to increased awareness of HAI as a real and relevant problem. To address the lack of knowledge regarding their own use of HH and AT, the participants talked about the discrepancy between self-estimated adherence and the observed adherence rates. Most participants expressed both disbelief and embarrassment: *“How was this measured?”*, *“This is truly terrible”*, and *“Unbelievably low adherence rates”*. However, it was not enough to acknowledge the low use of HH on a group level. The real turning point for the Lab participants was after the first Lab, when they were assigned to observe their own and their colleagues’ practice:

*“Now that I see* [the lack of HH]*, I cannot un-see.”*

*“Now I see it all the time, and it makes me frustrated.”*

From being a peripheral problem caused by people other than themselves, the lack of HH became central. The will to change was enacted, and the observations gave rise to many ideas for improving practice. For nurse anesthetists, the need to maintain the safety of patients was ever-present in their daily work, but the lack of HH and AT was not previously viewed as a safety issue. During the intervention there was a slow shift in perspective and a growing interest in finding solutions to address the inadequacies.

#### Co-creation, situated learning and iterative prototyping resulting in mind-changing turning points

The co-creation of standard operational procedures (SOP) for different invasive procedures became the core structure around which co-creative activities in the Lab and the OR evolved. The SOPs became a way of addressing the “My five moments of Hand Hygiene” which were not fully applicable to the situations in the OR. By co-creating SOPs the necessity of teamwork and communication during invasive procedures to avoid transmission of microorganisms were addressed and managed. Typically, every SOP was tested, refined, and reflected on in several cycles, producing at least four prototypes before a final version was agreed upon by the participants and the OR staff. This process took longer than participants had anticipated, and initially they found it difficult to understand and appreciate the explorative method. In many instances participants often said *“Just tell us what to do”* – a paradoxical statement, as top-down decisions were seldom well received. However, after some time the Lab participants came to appreciate the iterative process, as they felt that their colleagues became less reluctant and defensive about change if their knowledge and expertise were taken into account when refining the SOPs. This realization was a major turning point, particularly when mirrored against an unsuccessful attempt to create and implement another SOP. Some of the Lab participants had identified another area in the ward in need of improvement, and in their eagerness to find quick solutions, nurses and managers made decisions in haste. The SOP was implemented after just one informational meeting with the nursing staff, without using a co-creative approach. This new SOP attracted much critique, and even hostility and personal accusations from some staff. There were multiple reasons for this. First, the SOP and the change were not seen as meaningful by the staff. Second, in this particular example, the initiator did not view coworkers as partners in the change process, and so omitted the important steps of co-creation and meeting resistance to change with curiosity and respect. One participant expressed what they had learned:

*“It takes more time to work in this way* [co-creation and prototyping]*, but it’s worth it because change comes so much easier.”*

#### A growing awareness of the workplace culture and one’s own practices

During the year of the intervention, the participants became aware of their own basic assumptions regarding safety and risk in the OR, and how their own professional subcultures and the leadership co-constructed the working climate in the ward. They also became aware of the ways in which they spoke to others, about themselves and about other professional groups that either enabled a new understanding or cemented their stereotypical understandings of each other.

#### Increased psychological safety through dialogue

The Lab dialogue evolved over the implementation period, and the four phases described by Isaacs [45] were also identified in this study. In the first phase, the Labs were unstable, the underlying issues concerned safety and trust in the learning container, and the underlying emotional content involved scapegoating, disbelief, and shame. The second phase was defined by a search for order within the container, a search for how to communicate, and reflections over the process itself. In this stage, the underlying emotions were anxiety, anger, and hope. In the third phase, the group developed an internal stability, and trust and confidence in each other started to grow. Nevertheless, there was a tendency to talk about themselves in a different way and in contrast to “the others”, meaning the OR staff. Frustration, disappointment, and joy were dominant underlying emotions. The fourth phase was defined by new perceptions of self and others, along with insights on how practices could be transformed through cooperation and co-creation. In this phase, there was willingness for and growing insight into the necessity to include others in the group to increase involvement in sustaining the perceived changes in practice.

## Discussion

Our findings describe the complexity of implementing a seemingly simple preventive innovation involving AT and HH, and shows how organizational factors and strong professional subcultures can impede implementation efforts if not addressed. In this study, we have identified barriers towards changes in HH and AT behaviors in the OR context that reflect previous literature from general hospital settings [29, 30, 52] but also previously not described barriers as lack of psychological safety, learning anxiety and the belief that HH do not prevent postoperative infections.

Below, we discuss the central findings in relation to what worked as a mechanism for change and the active ingredients in our KT program: *agile and relational facilitation*, *creating a shared sense of urgency and intention*, *interprofessional dialogue*, and *iterative co-creation*.

### Agile and relational facilitation

The use of a learning container and facilitated dialogue was effective in temporarily closing the gaps within hierarchies and promoting psychological safety within the group, thus creating an atmosphere that supported dialogue and learning. We found that pre-diagnosis of context and pre-planning of activities was useful, but more important was the facilitators’ iterative analyses and adaptations to emerging situations and conditions. The facilitators and participants necessarily had to handle complex and sometimes conflicting realities. We suggest that by facilitating communicative relationships within the organization, the participants moved towards an increased ability to manage complexity. In line with previous work [53], our participants engaged in the mindful process of facilitating the creation of trustful and constructive relationships that occurred through the intervention. This process was vital to bridge the challenges formed by contextual and complexity aspects.

### Creating a shared sense of urgency and intention

The implementation of evidence-based knowledge in routine practice is often difficult [54–56]. According to Rogers, implementing a preventive innovation like HH is particularly challenging [57]. It is conceivable that the negative consequences of low adherence to HH guidelines or failure to use AT will not affect all patients. Moreover, when patients are affected, the symptoms of infection are not visible to care providers as the signs typically occur after patients have left the OR or even the hospital [58]. If patient outcome data are not regularly shared with the OR, this will lead to a false since of security and no motivation for change, as supported by our findings. Creating a shared sense of urgency and shared intentions among the participants inside and outside the Labs became a crucial aspect of creating a basis for implementation. Kotter [59] suggests that the inability to create a sense of urgency about a problem throughout the organization is one of the main contributing factors behind the failure of many change initiatives. Our findings support this assumption. However, in contrast to the early work by Kotter, we found that the process had to be iterative and cyclic rather than linear in order to create change. We used performance feedback [60], lectures [61], patient outcome data, and examples of in-hospital adverse events to start the process towards commitment to change. However, this alone was not enough to motivate change. It was important to consider how different professional subgroups perceived the level of evidence in support of the innovation, and to tailor the “packaging” and delivery of the information accordingly. Allowing for the free expression of different opinions and doubts and viewing resistance as a natural part of any change was effective in moving forward in the process [62]. Clearly more time would have been necessary to reach the physicians outside the Labs to fully create a shared sense of urgency and commitment toward changing HH and AT practices. The physician and front-line physician leaders were involved early and assigned to have the role as “change agents”. Our findings demonstrate that if the change agent is junior physician or for some other reason lower ranked with in the professional hierarchy, the ability to influence peers seems to be limited. This seems to be especially relevant if the implementation “object”, like AT, are perceived as a non-problem or a peripheral issue by the group.

### Interprofessional dialogue

Change is often characterized by uncertainty, and in a process where basic assumptions are challenged, anxiety might arise among the participants. In our study, the facilitators’ ability to understand and manage fear and anxiety was central. According to Schein [46], “learning anxiety” combines several specific fears, all of which may be active at any given time as one imagines having to unlearn something and learn something new*.* Loss of professional identity and group membership were relevant issues that confronted physicians. To willingly engage in discussions about HH and AT with one’s peers demands courage, as the discourse and knowledge around infection control is closely connected to professional identity. Thus, talking about HH and engaging in collaborative problem solving was equivalent to putting individuals in a risky situation with the potential to lose status and the important acceptance of the group. Edmondson suggests that these types of activities involve interpersonal risk-taking [63]. The responses to learning anxiety can be defensive and include denial of the problem, scapegoating, or the invention of various excuses for why health professionals cannot truly participate in a transformative learning process [64]. We observed these various responses during delivery of the intervention. The remedy to anxiety associated with learning is to increase the learner’s sense of psychological safety [46]. We used the Labs and facilitated interprofessional dialogue as a way to increase psychological safety.

### Iterative co-creation for bringing about relevant and meaningful change

Motivation was important, but insufficient in itself to bring about new practices. Enabling learning and innovation by creating an environment that supported and enabled the human capacity and innovation within the organization was crucial. By involving three professional groups, four sub-specializations, and managers, the participants were given an opportunity to interact in a different way through the Lab format. Working with the Lab during the course of a year enabled the building of person-centered relations that could cross the barriers of hierarchies and distrust and start to create new understandings of each other’s realities, and thus influence the culture.

The mechanisms of change were clearly embedded in the co-creation processes. We found that co-creation of SOPs and other activities between managers, leaders, and all the professionals involved in the OR was an essential component in our KT program. Co-creation has been defined as the development of a “shared body of usable knowledge” across scientific, governance, and local practice boundaries [65]. It is a way to involve different stakeholders that can jointly identify all dimensions of an issue. Our data support working with co-creation between nurses, physicians, managers, and researchers to ensure that the “product” becomes meaningful and relevant in relation to the context and the users. By using an iterative prototyping process, participants outside the Lab were constantly involved in the co-creative process of testing, reflecting, and refining the SOPs. This was essential to engage and use the expertise of the larger group. The process is time consuming, but by the time that a SOP has been finalized, the new practice has been implemented into everyday work.

## Strengths and limitations

The study has several limitations. Firstly, it is always difficult to understand what is really happening in a workplace. The artifacts of the OR and the espoused values are easily captured during participant observations and informal talk, but gaining deeper insights into the professional subcultures and the taken-for-granted assumptions that guide the behaviors of OR staff is truly difficult [46]. We do not claim that we have been able to capture the “true essence” of this workplace, but we have identified contextual and cultural factors useful for future implementers in this context. Another limitation was that the lack of continuity in the Labs affected the ability to work effectively during the process; nevertheless, the KT program was able to have a fundamental impact on how the participants talked about HAI, HH, and change. Implementing this KT program was time-consuming, and further studies are needed to investigate cost effectiveness and impact on actual adherence to HH and AT guidelines.

Several measures were taken to achieve trustworthiness during the data collection and analysis, such as regular discussion regarding the researchers’ pre-understandings and the potential impact this might have on interpretation, careful selection of meaning units in line with the intended focus of the study, and seeking agreement among co-researchers regarding thematic dimensions. To enhance credibility, the results were supported by representative quotations. Using an ethnographic approach in data collection enabled a deeper understanding of how different subcultures perceive a KT process and interact within it.

## Conclusions

Enabling nurses and physicians to think and talk differently about HAIs and HH requires a shift from the concept of one-way directed compliance towards change and learning as the result of a participatory and meaning-making process. Building capacity for co-creation involves strengthening relationships and communication between professional subgroups and managers and creating platforms for learning that cross the boundaries of departments and hierarchies. Our study shows how this is accomplished through four interrelated activity concepts: agile facilitation, creating a shared sense of urgency and intention, interprofessional dialogue, and iterative co-creation.

## Abbreviations

AT: Aseptic techniques
HAI: Hospital acquired infections
HH: Hand hygiene
KT: Knowledge translation
Labs: Learning Laboratories
OR: Operating room
SOP: Standard operational procedure
